# The Impact of AI-Assisted Learning on the Agency of Foreign Language Learners: A Meta-Analysis

**DOI:** 10.3390/bs16030379

**Published:** 2026-03-06

**Authors:** Fengyu Zai, Xiaoyong Zhou

**Affiliations:** School of Foreign Languages, East China Normal University, Shanghai 200241, China

**Keywords:** AI-assisted learning, foreign language learners, learner agency, meta-analysis

## Abstract

This study employs a meta-analytic approach to synthesize empirical evidence on the impact of AI-assisted learning on the agency of foreign language learners. The overall synthesis indicates positive associations between AI-assisted learning and multiple dimensions of learner agency; however, the magnitude of these associations varies substantially across studies. Among these outcomes, engagement shows the largest pooled effect size (r = 0.648), whereas enjoyment demonstrates the smallest (r = 0.392). Due to extreme heterogeneity, these pooled estimates serve only as descriptive summaries of the literature rather than evidence of robust effects, as variability significantly constrains their interpretability. Moderator analyses and meta-regression were conducted to explore potential sources of this heterogeneity. Although subgroup analyses reveal that neither learners’ first-language background, educational level, nor AI tool type significantly accounts for between-study variability, indicating that contextual factors likely shape outcomes in complex ways. These findings underscore the robust potential of AI-assisted learning while emphasizing the importance of investigating specific conditions—such as task design, teacher intervention, and learner profiles—that optimize agency development. Future research should move beyond global effect estimates toward context-sensitive strategies for maximizing AI’s impact on learner agency.

## 1. Introduction

With the rapid advancement of artificial intelligence (AI), AI-assisted foreign language teaching has emerged as a major innovation in contemporary education. Rather than functioning merely as a substitute for traditional instructional tools, AI technologies are increasingly embedded within pedagogical processes, reshaping how instruction, feedback, and learning support are delivered (Li & Towne, 2025). In foreign language education, AI applications—such as intelligent speech evaluation, automated writing feedback, and learning analytics—enable more adaptive instructional practices and data-informed pedagogical decision-making (Y. Zhang & Wang, 2020). As a result, AI-assisted learning has been widely associated with improvements in instructional efficiency and individualized learning outcomes.

AI-assisted learning in language education encompasses a broad range of technologies, including intelligent tutoring systems, speech recognition, machine translation, automated feedback tools, and analytics-driven platforms. These technologies are designed to support learners by adapting content, pacing, and feedback to individual needs, thereby reshaping learners’ engagement with language learning tasks (Mohebbi, 2024). Prior research has shown that such systems can facilitate learner control over learning processes, particularly through adaptive task sequencing and timely feedback mechanisms (Gill et al., 2023). However, most existing studies have primarily examined these technologies in relation to performance outcomes, engagement, or motivation, rather than learners’ agentic involvement in the learning process.

Learner agency has long been recognized as a central construct in foreign language learning, referring to learners’ capacity to actively regulate their goals, behaviors, and strategies within specific learning contexts (Emirbayer & Mische, 1998). Learners with stronger agency are more likely to engage in self-regulation, persist in the face of challenges, and strategically manage their learning processes. Although recent studies suggest that AI-assisted environments may foster agency by supporting self-regulation and adaptive learning behaviors (Tapalova & Zhiyenbayeva, 2022; Zawacki-Richter et al., 2019), empirical findings remain fragmented and conceptually diffuse.

Importantly, existing syntheses of AI in language education—including systematic reviews, bibliometric analyses, and meta-analyses—have largely focused on learning achievement, engagement, motivation, or technological affordances, while treating agency-related constructs as secondary or implicit outcomes (Wei, 2023; G. Xu et al., 2025; Wah, 2025). Importantly, learner agency is increasingly conceptualized not as a single psychological trait, but as a multidimensional construct encompassing learners’ proactive regulation of cognition, emotion, and behavior in context (Gao, 2010; Reeve & Tseng, 2011). Accordingly, agency-related outcomes such as autonomy, engagement, motivation, self-efficacy, enjoyment, and willingness to communicate represent interconnected yet theoretically distinct dimensions through which agency is enacted in foreign language learning environments. Therefore, rather than treating agency as a unitary variable, the present meta-analysis synthesizes evidence across these complementary dimensions to capture the broader impact of AI-assisted learning on learner agency.

At the same time, the impact of AI-assisted learning on learner agency is not uniformly positive. Over-reliance on AI-generated guidance may constrain learners’ independent exploration and critical engagement, potentially undermining agency development (L. Zhang & Xu, 2024). In addition, generative AI systems may exhibit cultural and linguistic limitations, leading to uneven learning experiences across learner populations (Godwin-Jones, 2024). These concerns highlight the need for a more nuanced and evidence-based understanding of how AI-assisted learning shapes learner agency.

To address these gaps, the present study conducts a meta-analysis of empirical research to systematically examine the effects of AI-assisted learning on the agency of foreign language learners. By synthesizing quantitative evidence across multiple agency dimensions and exploring moderating factors such as first language and educational level, this study extends prior reviews by offering a focused, statistically grounded analysis of learner agency. In doing so, it aims to clarify the magnitude, variability, and contextual conditions under which AI-assisted learning influences agency in foreign language education. To achieve this, the present study addresses the following research questions: (1) To what extent does AI-assisted learning affect the agency of foreign language learners? (2) How do specific dimensions of agency—autonomy, engagement, motivation, self-efficacy, enjoyment, and willingness to communicate—differ in their response to AI-assisted learning? (3) What factors may moderate the impact of AI-assisted learning on learners’ agency?

## 2. Literature Review

### 2.1. The Agency of Foreign Language Learners

Agency refers to an individual’s ability to actively regulate their own behaviors, motivations and learning strategies within a specific context (Emirbayer & Mische, 1998). The agency of foreign language learners is rooted in social cognitive theory and self-determination theory (Bandura, 2001; Deci & Ryan, 1985), emphasizing learners’ proactivity, autonomy and self-regulation in the learning process. Agency not only pertains to whether learners adopt a proactive learning attitude but also involves how they regulate their emotions, motivations and cognitive strategies to adapt to different learning situations (Gao, 2010).

Specifically, agency encompasses several core dimensions, including goal setting, selection of learning strategies, adaptation to the learning environment and positive feedback on learning outcomes (Reeve & Tseng, 2011). Goal setting involves learners’ ability to establish clear learning objectives and dynamically adjust them throughout the learning process. The choice of learning strategies reflects whether learners can effectively utilize cognitive, metacognitive and social interaction strategies to enhance learning outcomes. Adaptation to the learning environment is manifested in how learners adjust their behaviors in different educational contexts (such as traditional classrooms, self-directed learning, or AI-assisted environments) to maximize learning gains. Positive feedback on learning outcomes emphasizes learners’ ability to use external feedback and self-assessment to adjust their learning approaches, thereby enhancing their confidence and learning effectiveness.

Research indicates that highly agentic learners tend to employ deeper learning strategies, such as critical thinking, reflective learning and autonomous inquiry, leading to stronger self-regulation in foreign language learning (Zimmerman, 2000). Furthermore, these learners often maintain higher levels of motivation throughout the learning process, demonstrating resilience even in the face of challenges. Empirical evidence from diverse educational contexts—including European and North American settings—suggests that learner agency is positively associated with sustained engagement, strategic learning behavior, and long-term language development, particularly in learner-centered and technology-enhanced environments (Reinders & Benson, 2017; O’Dowd, 2018). These characteristics enable highly agentic learners to exhibit greater persistence and higher levels of language acquisition during the foreign language learning process.

### 2.2. The Application of AI-Assisted Learning in Foreign Language Teaching

In recent years, the application of AI technology in foreign language teaching has become increasingly widespread. Adaptive learning systems can provide personalized learning content based on learners’ individual differences, intelligent writing feedback systems offer instant language corrections and improvement suggestions and speech recognition technology helps learners enhance their pronunciation accuracy (Bourekkache et al., 2017). Additionally, AI-based learning analytics can track learners’ behavioral patterns, providing data support for teachers to optimize their teaching strategies (Salas-Pilco et al., 2022). The use of these AI tools has improved the interactivity and flexibility of learning, creating a more personalized learning environment for foreign language learners.

Moreover, AI applications in foreign language teaching also include intelligent chatbots, virtual reality (VR) and augmented reality (AR) technologies and automated scoring systems. Intelligent chatbots, such as ChatGPT, can provide instant conversation practice, allowing learners to apply their knowledge in real contexts. VR and AR technologies simulate immersive scenarios, enabling learners to interact in realistic language environments and enhancing their language proficiency (Gill et al., 2023; X. Qiu et al., 2021). Furthermore, AI-driven automated scoring systems can quickly assess writing and speaking tasks while providing detailed feedback to help learners continuously improve (Alharbi, 2023; AbuSahyon et al., 2023).

Beyond East Asian and Middle Eastern contexts, studies conducted in Western and Latin American settings have also reported positive effects of AI-assisted language learning on learner engagement, self-regulation, and communicative competence, particularly in blended and online learning environments (Kohnke & Moorhouse, 2022; Urzúa et al., 2025). These applications of AI not only enhance the level of personalization in learning but also make teaching more data-driven. Through learning analytics, AI can record learners’ progress, identify their weaknesses and recommend targeted learning content. This data-driven teaching model helps teachers formulate more precise teaching plans, thereby optimizing teaching effectiveness. At the same time, the proliferation of AI technology has made remote education and blended learning models more efficient, providing broader learning opportunities for learners from diverse backgrounds.

However, despite the numerous advantages of AI technology in foreign language teaching, its limitations should not be overlooked. For example, automated scoring systems still struggle to fully understand complex language expressions, which may lead to biased assessment results (Fagbohun et al., 2024). More critically, recent empirical studies have highlighted the risk of over-reliance on AI systems, whereby excessive dependence on automated feedback and content generation may reduce learners’ metacognitive engagement and self-initiated problem-solving behaviors (Zhai et al., 2024; Creely, 2024). Additionally, over-reliance on AI may deprive learners of opportunities for active thinking and exploration, thereby affecting the development of their critical thinking skills (Dif & Bousioud, 2024). Therefore, the application of AI in foreign language teaching needs to be integrated with traditional teaching methods to ensure that learners can enjoy the conveniences brought by technology while maintaining the necessary agency and autonomy in their learning.

### 2.3. AI-Assisted Learning and the Agency of Foreign Language Learners

Whether the introduction of AI technology can effectively enhance the agency of foreign language learners is still a question worth exploring. On one hand, the personalized feedback and adaptive learning paths provided by AI may play a crucial role in boosting learners’ self-efficacy, thereby enhancing their capacity for autonomous learning. Research has shown that when learners receive timely feedback and have control over their learning pace in adaptive learning environments, they are more likely to exhibit higher levels of agency and engage more proactively in the learning process (Clark, 2012). For instance, intelligent language learning tools, such as Duolingo and ChatGPT, create real-time interactive opportunities for learners. These platforms allow learners to practice and apply their target language within immersive environments, which not only enhances their learning confidence but also significantly boosts their motivation to engage with the material (Martunyuk, 2024). The ability to interact dynamically with content reinforces the idea that learners have agency in their educational journeys, encouraging them to take active roles in their learning experiences.

From a conceptual perspective, different AI tools appear to support distinct components of learner agency (Mouta et al., 2025; Xia et al., 2025). For example, adaptive learning systems are closely linked to autonomy through self-paced learning and task selection, while conversational AI tools such as ChatGPT are more strongly associated with motivation and willingness to communicate by lowering interactional anxiety and increasing practice opportunities (Wah, 2025). Automated feedback systems, in turn, primarily influence self-efficacy and engagement by providing immediate performance-related information (Y. Qiu & Ishak, 2025).

However, on the other hand, an over-reliance on AI tools may inadvertently diminish learners’ agency, rendering them passive recipients of knowledge (Creely, 2024). If learners become accustomed to depending on AI for answers and solutions, they may forgo opportunities for independent exploration and the development of critical thinking skills (Dif & Bousioud, 2024). Empirical studies have shown that when AI-generated suggestions are accepted uncritically, learners’ reflective processing and strategic decision-making are significantly reduced, indicating a potential erosion of agentic control (Zhai et al., 2024). Moreover, although AI recommendation systems can offer personalized learning pathways based on learners’ behavioral data, they may also restrict exposure to a wider array of learning resources, potentially hindering creativity and independent learning (Dhananjaya et al., 2024).

This nuanced relationship indicates that different types of AI tools and their specific applications can significantly impact learners’ agency in varying ways. Despite a growing body of empirical studies, existing research remains fragmented, with most studies examining isolated agency-related variables rather than agency as an integrated, multidimensional construct. In this study, six dimensions—autonomy, engagement, motivation, self-efficacy, enjoyment, and willingness to communicate—were selected to represent learner agency. These variables were chosen because they encompass the cognitive, affective, and behavioral components that are central to the exercise of agency in second language acquisition (Gao, 2010; Reeve & Tseng, 2011). While motivation and engagement are frequently researched, their inclusion alongside constructs like autonomy and self-efficacy allows for a more comprehensive assessment of how learners proactively navigate AI-enhanced environments. Therefore, it is essential to conduct a thorough analysis of how AI-assisted learning affects foreign language learners’ agency. This research aims to systematically integrate and analyse existing empirical studies on the impact of AI-assisted learning on learners’ agency while exploring associated influencing factors. By synthesizing quantitative findings across multiple agency dimensions and AI tool types, the present meta-analysis seeks to clarify both the benefits and potential constraints of AI-assisted learning for learner agency.

## 3. Methodology

### 3.1. Literature Search

This study conducted a search in five English databases (Web of Science, JSTOR, Educational Resource Information Center, ProQuest, Wiley Online Library) for literature that includes AI-assisted learning and foreign language achievement in the title or abstract. The search keywords were integrated using Boolean logic operators as follows: (large language models OR LLMs OR artificial intelligence OR generative AI OR AI OR gen-AI) AND (second language OR foreign language OR second language acquisition OR foreign language acquisition OR second language learning OR foreign language learning OR SL OR FL) AND (agency OR autonomy OR self-direction OR self-efficacy OR motivation OR engagement OR enjoyment OR willingness to communicate) AND (learner OR student).

### 3.2. Inclusion Criteria

Research that met our inclusion criteria underwent a review of titles and abstracts and all studies containing specific terms were included in this review. Given that the primary language used in our databases is English, studies in other languages were also considered. To be included in our review, papers had to meet the following eligibility criteria:

(1) The article must report primary research; commentaries and reviews are excluded.
(2) The articles must have been published between 2016 and 2026.
(3) The articles must employ either a quantitative or a mixed-methods design with a significant quantitative component.
(4) The article must contain complete data.
(5) The article should focus on language learners or students.
(6) The article should focus on AI-assisted learning.
(7) At least one of the research questions must investigate the impact of AI-assisted learning on the agency of language learners.

### 3.3. Exclusion Criteria

In addition to the inclusion criteria, we established specific exclusion criteria to ensure that only relevant and high-quality studies are reviewed:

(1) Studies that do not report primary research, such as commentaries, theoretical papers, or reviews, will be excluded.
(2) Articles published before 2016 or after 2026 were not considered.
(3) Studies that either solely use a qualitative approach or employ a mixed-methods approach without a significant quantitative component will be excluded.
(4) Articles with incomplete or missing data were not included in our analysis.
(5) Studies not focusing on language learners or students as participants were excluded.
(6) Studies not focusing on AI-assisted learning as the main research content were excluded.
(7) Articles that do not specifically explore the impact of AI-assisted learning on language learners’ agency are omitted.

### 3.4. Selected Studies

**Figure 1:**
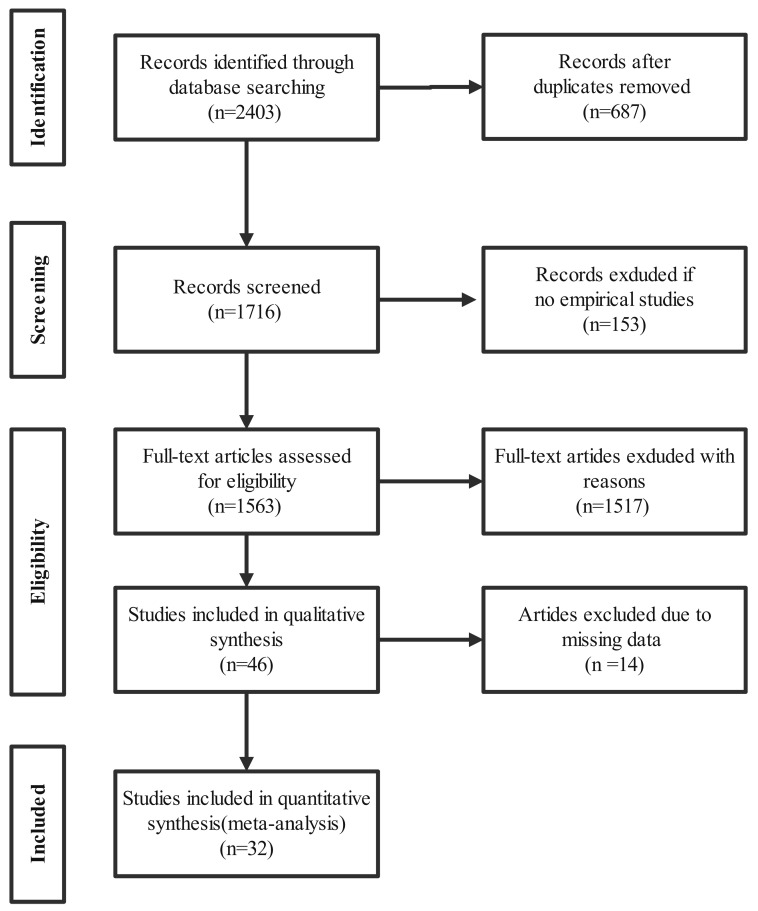
PRISMA flowchart for the identification, screening and inclusion of publications in the meta-analyses.

A systematic literature search was performed, followed by duplicate removal and multi-stage screening. After full-text assessment based on the predefined inclusion criteria, 32 studies were included in the final analysis (see Figure 1). To minimize selection bias, full-text screening and data coding were conducted independently by two researchers. During the coding process, conceptually overlapping variables were consolidated to ensure comparability across studies. For example, academic engagement, social engagement, cognitive engagement, emotional engagement, affective engagement, and behavioural engagement were all coded under the unified category of “Engagement.” Coding discrepancies were resolved through consensus discussions, ultimately achieving an acceptable level of agreement.

### 3.5. Statistical Analysis

The information on authors, publication year, participants, first language, AI Type, correlation coefficients and sample size was coded (see Table 1). This study used the Pearson correlation coefficient (r) as the effect size and the extraction of effect values followed these principles: (1) Effect values were extracted and coded as independent samples; if the same study investigated multiple independent samples, they were coded separately; (2) If the study did not report the correlation coefficient, it was converted to an r value using the formulas provided by Schmidt and Hunter (2004) before coding.

To address substantial heterogeneity, a random-effects meta-regression was conducted using AI tool type as a moderator. AI tools were coded based on their primary pedagogical function and analyzed using Fisher’s Z–transformed effect sizes in Comprehensive Meta-Analysis (CMA). The meta-analysis employed the DerSimonian and Laird (1986) method to estimate effect sizes. The Q test was typically used to assess heterogeneity, evaluating whether the effect sizes across studies were homogenous. The Q test is sensitive to the number of studies, meaning that the Q value increases with the degrees of freedom. The significance level for the Q test was generally set at α = 0.10, with *p* < 0.10 indicating the presence of heterogeneity among the studies. The existence of heterogeneity necessitated the use of a random effects model in the meta-analysis. The articles included in the meta-analysis demonstrated a closed association between the target variables. The overall sample consisted of 6558 participants. The inclusion criteria for the study encompassed the first language of the participants, with Chinese being the most frequently reported first language in the reports (21 studies).

### 3.6. Publication Bias

Publication bias refers to the phenomenon where studies with significant results are more readily accepted and published, making it difficult to obtain research that does not yield significant results during the literature collection process, thus affecting the accuracy of meta-analysis results (Rothstein, 2005). In this study, in addition to including published journal articles and conference papers, efforts were made to obtain unpublished theses, which helped mitigate publication bias to some extent. Furthermore, during the meta-analysis process, various methods (funnel plots, Egger’s regression) were employed to test for publication bias. For funnel plots, if the graph is symmetrical, it indicates a smaller publication bias and a lesser impact on the meta-analysis results (Light & Pillemer, 1984); regarding Egger’s regression, if the results of the linear regression are not significant, it suggests a smaller publication bias (Egger et al., 1997).

## 4. Results

### 4.1. Heterogeneity Test

The results of the heterogeneity test indicate that (see Table 2), the heterogeneity analysis revealed a Q value of 2325.001 (*p* < 0.001) and an I^2^ value of 96.99%, exceeding the 75% threshold for high heterogeneity proposed by Huedo-Medina et al. (2006). This suggests that the effect sizes exhibit high heterogeneity. The estimated τ^2^ value (0.148) further indicates substantial between-study variance, justifying the use of a random-effects model and subsequent moderator analyses.

### 4.2. Publication Bias Test

**Figure 2:**
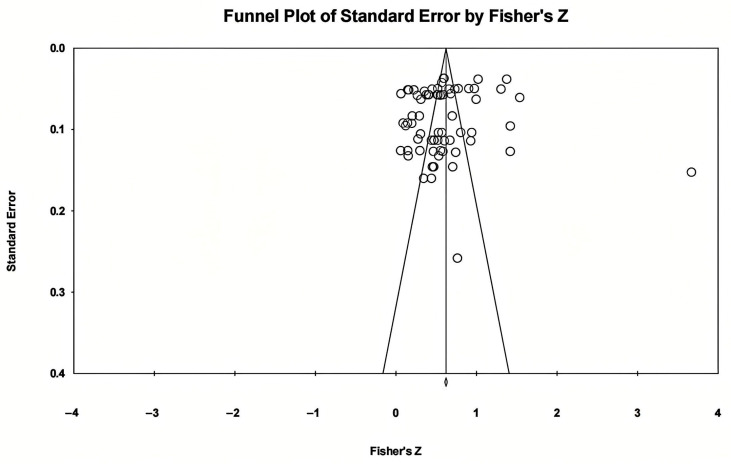
Funnel chart results.

**Figure 3:**
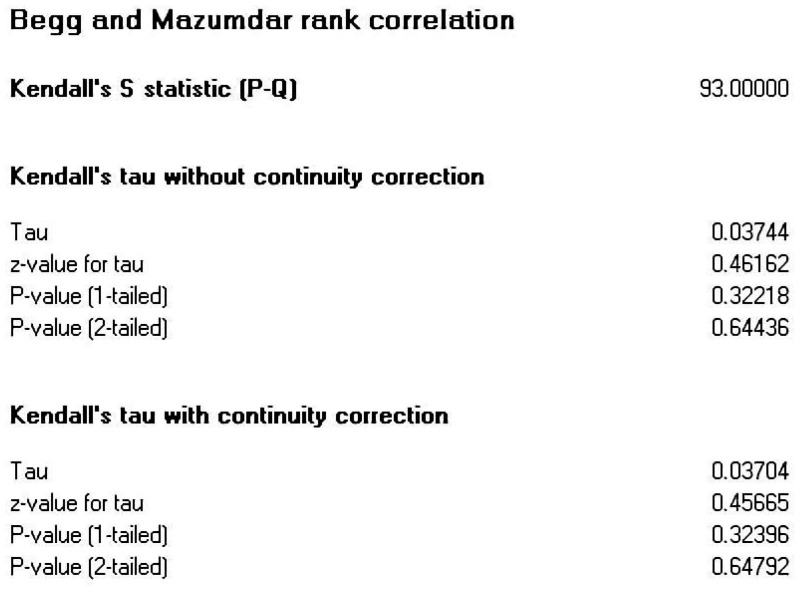
Kendall’s S statistic rank correlation.

**Figure 4:**
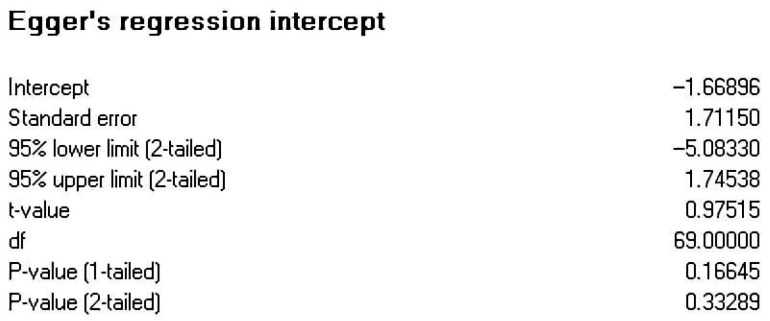
Egger’s regression intercept.

The funnel plot (see Figure 2) shows that the effect sizes of AI-assisted learning and agency in foreign language learners are mostly located above the funnel plot and are evenly distributed on both sides of the central line. The rank correlation analysis revealed no significant difference (z = 0.457, *p* = 0.647) (see Figure 3) and Egger’s regression test also indicated no significant difference (t = 0.975, *p* = 0.332) (see Figure 4). These nonsignificant results indicate symmetry and suggest an absence of publication bias. The above results indicate that this research does not exhibit significant publication bias.

To examine the robustness of the pooled estimates, we conducted a sensitivity analysis by excluding extreme outlier effect sizes (e.g., r > 0.90). The results indicated that the direction of association remained positive, although interpretability remains constrained by substantial heterogeneity. This suggests that the main conclusions of the meta-analysis were not driven by a small number of unusually large effect sizes.

### 4.3. Subgroup Analysis

#### 4.3.1. Autonomy

As shown in Table 2, the effect of AI-assisted learning on autonomy is positive, with an effect size of r = 0.581 (95% CI [0.305, 0.767], *p* < 0.001). This subgroup includes a total of 7 studies (K = 7). The Q value is 233.491, and the high I^2^ value of 97.43% indicates substantial heterogeneity within this subgroup. These findings suggest that although the pooled estimate is statistically significant, the magnitude of the effect varies considerably across studies, suggests that the strength of this effect is not consistent across all studies. Variations in instructional design and the degree of learner control embedded within AI tools may partially account for this variability.

#### 4.3.2. Engagement

As shown in Table 2, the effect of AI-assisted learning on engagement is positive, with an effect size of r = 0.648 (95% CI [0.476, 0.773], *p* < 0.001). This subgroup includes 15 studies (K = 15). The Q value is 634.252, with a high I^2^ value of 97.79%, indicating substantial heterogeneity among studies. Engagement demonstrated the largest pooled effect size among all dimensions. At the same time, the substantial heterogeneity within this subgroup indicates that engagement outcomes differ markedly across implementations. Differences in tool interactivity, task type, or duration of intervention may contribute to this dispersion.

#### 4.3.3. Enjoyment

As indicated in Table 2, the effect of AI-assisted learning on enjoyment is positive, with an effect size of r = 0.392 (95% CI [0.226, 0.535], *p* < 0.001). This subgroup includes 18 studies (K = 18). The Q value is 621.849, and the I^2^ value of 97.27% reflects considerable heterogeneity. AI-assisted learning also showed a positive effect on learner enjoyment. However, the high heterogeneity suggests that enjoyment is particularly sensitive to contextual and technological factors, such as interface design, task difficulty, or learners’ prior familiarity with AI tools.

#### 4.3.4. Motivation

According to Table 2, AI-assisted learning has a positive effect on motivation, with an effect size of r = 0.540 (95% CI [0.344, 0.691], *p* < 0.001) based on 13 studies (K = 13). The heterogeneity is substantial (Q = 515.555, I^2^ = 97.67%). The findings indicate that AI-assisted learning positively influences learner motivation. Given the high level of heterogeneity observed in this subgroup, motivational gains should be interpreted with caution, as they may depend on specific learner characteristics or pedagogical conditions.

#### 4.3.5. Self-Efficacy

As indicated in Table 2, the effect of AI-assisted learning on self-efficacy is positive, with an effect size of r = 0.510 (95% CI [0.447, 0.568]), based on 7 studies (K = 7). The Q value is 10.945, and the I^2^ value of 45.18% suggests relatively low heterogeneity compared with other dimensions. The overall effect approaches but does not reach conventional levels of statistical significance (*p* = 0.090).

#### 4.3.6. Willingness to Communicate

As shown in Table 2, AI-assisted learning has a positive effect on willingness to communicate, with an effect size of r = 0.557 (95% CI [0.418, 0.671], *p* < 0.001), based on 11 studies (K = 11). The Q value is 190.336, and the I^2^ value of 94.75% indicates substantial heterogeneity. The analysis revealed the positive relationship between AI-assisted learning and willingness to communicate. Nonetheless, the high heterogeneity indicates that communicative benefits may vary depending on how conversational or interactive the AI system is within different learning contexts.

The synthesized data suggests that AI-assisted learning has the potential to enhance learner agency; however, this effect is far from consistent and varies significantly depending on specific contexts and pedagogical conditions. The overall heterogeneity was exceptionally high (I^2^ = 96.99%), indicating that the observed variability across studies far exceeds what would be expected from sampling error alone. Under such conditions, a single pooled effect size cannot be assumed to represent a meaningful or generalizable population effect. Accordingly, the pooled estimate is best understood as a descriptive summary of a highly heterogeneous body of research rather than as evidence of a consistent or robust impact of AI-assisted learning on learner agency.

### 4.4. Moderating Variable Analysis

#### 4.4.1. First Language

##### Chinese

According to Table 3, the effect of AI-assisted learning on learner agency among Chinese learners is positive, with a pooled effect size of r = 0.505 (95% CI [0.429, 0.574]). This subgroup comprises 52 studies (K = 52), representing the largest body of evidence in the dataset. The heterogeneity statistics indicate substantial variability across studies (Q = 1373.427, df = 51, *p* < 0.001, I^2^ = 96.287%), with a τ^2^ value of 0.119.

##### Persian

According to Table 3, AI-assisted learning demonstrates a strong positive effect on learner agency among Persian learners, with a pooled effect size of r = 0.814 (95% CI [0.612, 0.916]) based on 7 studies (K = 7). The heterogeneity within this subgroup is extremely high (Q = 583.257, df = 6, *p* < 0.001, I^2^ = 98.971%), accompanied by a τ^2^ value of 0.325.

##### Korean

For Korean learners, the pooled effect size of AI-assisted learning on learner agency is positive (r = 0.427, 95% CI [−0.066, 0.752]), based on 4 studies (K = 4). The heterogeneity is high (Q = 86.608, df = 3, *p* < 0.001, I^2^ = 96.536%), with a τ^2^ value of 0.274. Although the point estimate suggests a positive association, the wide confidence interval and substantial heterogeneity indicate that these results should be interpreted with caution.

##### Japanese

As indicated in Table 3, AI-assisted learning shows a positive effect on learner agency among Japanese learners, with a pooled effect size of r = 0.378 (95% CI [0.098, 0.603]) derived from 3 studies (K = 3). The heterogeneity statistics reveal considerable variability (Q = 20.224, df = 2, *p* < 0.001, I^2^ = 90.111%), with a τ^2^ value of 0.063. While the overall effect is statistically significant, the limited number of studies suggests that the findings provide preliminary evidence regarding the role of AI-assisted learning in Japanese EFL contexts.

##### Other Languages

The analysis included several first-language contexts with limited representation. For Kurdish learners, the meta-analysis revealed a positive pooled effect size (r = 0.355, 95% CI [−0.230, 0.752]) derived from two studies (K = 2). However, the statistical heterogeneity was exceptionally high (Q = 60.846, df = 1, *p* < 0.001, I^2^ = 98.357%), accompanied by a τ^2^ value of 0.188. Given the paucity of studies and the resulting wide confidence interval, these findings should be regarded as descriptive and tentative rather than definitive evidence. In addition to the Kurdish subgroup, individual studies provided data for three other linguistic contexts: Kazakh, Pakistani, and Arabic. Because these subgroups consisted of only a single study each, heterogeneity statistics could not be computed. As such, these values serve as preliminary indicators rather than robust inferential evidence. Ultimately, the significant imbalance in first-language representation underscores a systemic gap in the literature.

#### 4.4.2. Educational Level

##### Elementary School

According to Table 4, the effect of AI-assisted learning on learner agency at the elementary school level is positive, with a pooled effect size of r = 0.444 (95% CI [0.113, 0.686]), based on 6 studies (K = 6). The heterogeneity analysis revealed substantial variability across studies (Q = 90.500, df = 5, *p* < 0.001, I^2^ = 94.475%), with a τ^2^ value of 0.194.

##### Secondary School

As shown in Table 4, AI-assisted learning demonstrates a positive effect on learner agency among secondary school learners, with a pooled effect size of r = 0.511 (95% CI [0.396, 0.610]) derived from 9 studies (K = 9). The heterogeneity within this subgroup remains high (Q = 110.654, df = 8, *p* < 0.001, I^2^ = 92.770%), although lower than that observed at the elementary level.

##### University

According to Table 4, for university-level learners, the pooled effect size of AI-assisted learning on learner agency is r = 0.546 (95% CI [0.465, 0.618]), based on 56 studies (K = 56). The heterogeneity statistics indicate very high between-study variability (Q = 2045.891, df = 55, *p* < 0.001, I^2^ = 97.312%), with a τ^2^ value of 0.165.

A random-effects meta-regression was conducted to examine whether education level moderated the effect of AI-assisted learning on learner agency, using Fisher’s Z as the dependent variable (see Table 5). Elementary school served as the reference category, and 71 effect sizes were included. The results showed that education level was not a significant moderator. Compared with elementary school learners, neither secondary school learners (β =0.096, SE = 0.2083, Z = 0.46, *p* = 0.3225) nor university learners (β = 0.1372, SE = 0.1714, Z = 0.8, *p* = 0.2117) demonstrated significantly different effect sizes. The omnibus test of moderators was also non-significant (Q = 0.68, df = 2, *p* = 0.7111). Substantial heterogeneity remained unexplained (I^2^ = 96.97%), and the model accounted for virtually no between-study variance (R^2^ analog = 0.01), indicating comparable effects of AI-assisted learning on learner agency across educational levels. Although the subgroup estimates differed descriptively across educational levels (Table 4), these differences should not be interpreted as moderation effects unless supported by meta-regression results (Table 5).

#### 4.4.3. Meta-Regression by AI Tool Type

To further explore potential sources of the extreme heterogeneity observed across studies, a random-effects meta-regression was conducted using AI tool type as a moderator (Table 6). Effect sizes were transformed to Fisher’s Z values prior to analysis. AI interventions were categorized according to their primary pedagogical function, and general AI-assisted tools were treated as the reference category.

The intercept, representing the average effect size for the general AI-assisted group, was statistically significant (β = 0.5783, SE = 0.1368, 95% CI [0.3101, 0.8465], *p* < 0.001).

However, comparisons between the reference group and specific AI subtypes revealed no statistically significant differences. In particular, the coefficients for AI chatbots (β = −0.0721, *p* = 0.3368), AI-IDLE (β = −0.0014, *p* = 0.4972), and AI-integrated learning platforms (β = 0.0631, *p* = 0.3391) were all non-significant. These findings suggest that the magnitude of agency-related outcomes does not vary substantially across different categories of AI tools when compared with general AI-assisted learning interventions.

The omnibus test of moderators further indicated that AI tool type did not significantly explain between-study variance (Q = 1.27, df = 3, *p* = 0.7372), and the model accounted for virtually none of the observed heterogeneity (R^2^ analog = 0.00). Substantial residual heterogeneity remained after including this moderator (I^2^ = 97.09%, τ^2^ = 0.1556), suggesting that variability in effect sizes is more likely driven by broader contextual and pedagogical factors—such as instructional design, learner characteristics, intervention duration, and measurement differences—rather than technological classification alone.

## 5. Discussion

This study utilized a meta-analysis approach to systematically integrate existing empirical research on the impact of AI-assisted learning on the agency of foreign language learners. Overall, the findings indicate that AI-supported environments can enhance learner agency, although the magnitude and nature of these effects vary substantially across contexts and dimensions. Among these dimensions, engagement shows the largest effect size; conversely, enjoyment has the smallest effect size, suggesting that AI tools may foster active participation more reliably than affective experiences. Studies show that learners with high agency are generally more effective in utilizing AI tools, leading to improved learning outcomes (Zimmerman, 2000). This is consistent with the perspectives of social cognitive theory and self-determination theory, which emphasize learners’ proactivity and self-regulation in the learning process (Bandura, 2001; Deci & Ryan, 1985). These results suggest that AI tools may create conditions that support agentic engagement in certain settings, but such support is neither automatic nor uniform.

A critical issue emerging from this meta-analysis is the extremely high level of heterogeneity observed across most agency dimensions, reflecting context-dependent variability rather than a uniform effect. Potential sources of this heterogeneity include differences in educational settings, types of AI tools, research designs, intervention duration, and measurement instruments used to operationalize agency-related constructs. To further examine whether technological differences contributed to this variability, a random-effects meta-regression was conducted with AI tool type as a moderator. The results indicated that AI tool type did not significantly predict effect size variation, and the proportion of between-study variance explained by this moderator was negligible, suggesting that categorical distinctions among AI technologies alone are insufficient to capture the pedagogical mechanisms underlying agency development. Moreover, substantial residual heterogeneity remained after accounting for tool type, highlighting that contextual and instructional factors likely play a more decisive role than technological category in shaping learner agency outcomes.

Notably, self-efficacy presents a distinctive pattern in this meta-analysis. Unlike other agency-related variables, the overall effect of AI-assisted learning on self-efficacy did not reach statistical significance (*p* = 0.090) and exhibited relatively low heterogeneity. The comparatively lower heterogeneity suggests that, although the overall effect was non-significant, the estimates were relatively consistent across studies. This finding contrasts with several meta-analyses in second language acquisition and educational technology research, which have reported moderate positive effects of digital interventions on learners’ self-efficacy (C. Wang & Sun, 2020; Yi et al., 2024). It also differs from the recent meta-analysis by Ren et al. (2026), which reported a large and significant effect of AI-supported learning on self-efficacy. One explanation may lie in construct stability and contextual specificity. Self-efficacy in foreign language learning may be less responsive to short-term technological exposure than more state-like constructs such as engagement or motivation. In addition, excessive reliance on AI-generated feedback may reduce learners’ opportunities to attribute success to their own effort and strategy use, thereby weakening the development of self-efficacy despite improved task performance (Zhai et al., 2024; Dif & Bousioud, 2024).

In the moderation variable analysis, the study found descriptive differences across learners’ first-language backgrounds, with larger pooled estimates observed in Persian and Chinese samples than in some other language groups. These descriptive patterns tentatively suggest that linguistic and cultural backgrounds may shape how learners engage with AI-assisted environments. However, several non-Chinese subgroups were based on very few studies, and substantial within-group heterogeneity remained, limiting strong inferences about language background as a systematic moderator. Regarding educational level, subgroup analyses showed descriptive variation in effect sizes across elementary, secondary, and university samples, but meta-regression results indicated that educational level was not a statistically significant moderator of AI-assisted learning effects on learner agency. Although descriptive variation was observed across educational stages, meta-regression did not identify educational level as a significant moderator. Substantial heterogeneity persisted within each category, suggesting that differences within educational levels were greater than differences between them. This pattern implies that instructional design features and learner–AI interaction structures may play a more decisive role than formal educational stage. This suggests that the agency-supporting potential of AI-assisted learning may be broadly comparable across educational stages, while substantial within-group variability remains. One possible reason is that agency-related outcomes are more influenced by instructional design and task affordances than by educational stage. This interpretation is further supported by the meta-regression findings, which indicate that neither educational level nor AI tool type alone sufficiently explains the observed heterogeneity. AI tools offering adaptive feedback, interactive practice, and learner control may have similar motivational and regulatory effects across age groups. Additionally, the significant residual heterogeneity indicates that within-group variability might be more important than between-group differences based on education level.

Importantly, several additional moderators were theoretically considered as potential sources of heterogeneity, including intervention duration and intensity, target language type (e.g., EFL vs. other foreign language contexts), study design characteristics (experimental vs. cross-sectional), and measurement instruments used to operationalize agency-related outcomes. During the coding process, we attempted to extract these variables; however, they were not consistently or sufficiently reported across the included primary studies. In particular, many studies did not provide clear information regarding the length or frequency of AI-assisted interventions, and several employed correlational or cross-sectional designs without a well-defined intervention period. Likewise, detailed descriptions of instructional implementation and outcome measurement were often incomplete, making reliable classification difficult. Therefore, these potentially important moderators could not be systematically examined in the present meta-analysis without introducing excessive assumptions or subjective coding.

The practical implications of this study should be considered exploratory and context-bound. In contexts where AI tools are pedagogically integrated with reflective scaffolding and learner autonomy supports, positive agency-related outcomes may be more likely to emerge. However, the present findings suggest that the effectiveness of AI tools depends less on their technological category and more on their pedagogical integration. For AI tool designers, the results suggest the importance of embedding reflection scaffolds, such as prompts for self-explanation, goal setting, and strategy evaluation, to prevent learners from passively accepting AI-generated outputs. For teachers, AI feedback should be deliberately combined with learner self-assessment and peer reflection activities, encouraging learners to critically evaluate feedback rather than relying on it unconditionally. Such pedagogical integration can help ensure that AI functions as a support for agency development rather than a substitute for learner decision-making.

However, this study also has several limitations. Despite including multiple studies, the overall sample size remains limited, particularly in certain languages (such as Kurdish and Arabic) and educational levels, where the number of relevant studies is scarce. This restriction may lead to inadequate representation of the results, limiting comprehensive understanding of learners’ agency across different languages and educational backgrounds. Although moderator analyses based on first language and educational level were conducted, these variables explained little between-study variance. Similarly, the meta-regression analysis showed that AI tool type accounted for only a negligible proportion of the observed heterogeneity, highlighting the structural complexity of AI-assisted learning research. With I^2^ values exceeding 95% for most agency-related dimensions, the assumption that a single underlying “true” effect exists is untenable. This reinforces the interpretation that the pooled estimates should be understood as descriptive summaries of a heterogeneous body of evidence rather than definitive population parameters. The persistence of extreme heterogeneity despite the application of random-effects modeling and subsequent moderator analyses suggests that the observed variability may stem from deeper structural differences across studies. However, due to differences in study design, some studies did not report these variables and therefore could not be analysed. Consequently, rather than asking whether AI-assisted learning is effective in enhancing learner agency, future research should prioritize understanding when, for whom, and under what pedagogical conditions AI-supported environments are more likely to foster agentic engagement. From this perspective, the present meta-analysis should be viewed as mapping the diversity and dispersion of effects in the literature, rather than establishing a definitive conclusion about effectiveness. In addition, this meta-analysis was unable to systematically control for the methodological quality of the included studies, such as variations in research design, measurement instruments, and intervention duration. Moreover, the dataset shows a clear over-representation of Chinese learners, which may bias the overall effect estimates and limit the generalizability of the findings to other cultural and educational contexts. Beyond methodological limitations, ethical considerations surrounding AI-assisted learning warrant attention. Although sensitivity analyses excluding extreme outliers yielded comparable results, future research with larger and more balanced datasets is still needed to provide more stable estimates across diverse contexts. Recent studies have highlighted potential risks related to learner surveillance, data privacy, algorithmic bias, and excessive dependence on AI-generated feedback (Zhai et al., 2024; Creely, 2024). If unregulated, these issues may undermine learners’ sense of control and autonomy, thereby posing a threat to agency development.

Future research should move beyond predominantly cross-sectional evidence by adopting longitudinal designs to examine how learner agency develops under sustained exposure to AI-assisted learning, thereby clarifying the long-term effects of AI use on learners’ autonomy and motivation. Rather than focusing on broad categories of AI tools, future studies should pay closer attention to specific instructional design features, learner–AI interaction patterns, and scaffolding mechanisms, as existing meta-regression findings suggest that tool typology alone cannot adequately explain differences in agency-related outcomes. In this regard, identifying which AI functions—such as adaptive feedback, gamification, or opportunities for social interaction—most effectively support agency development would provide more concrete guidance for AI-enhanced pedagogy. In addition, cross-linguistic, cross-cultural, and cross-platform comparisons are needed to explore how cultural, linguistic, and technological contexts moderate learners’ engagement with and adaptation to AI-assisted learning. Moreover, experimental research that integrates AI tools with traditional teacher-led scaffolding, guided reflection, and pedagogical support would offer clearer insights into how human–AI collaboration can foster active learner engagement while preventing over-reliance on technology. By jointly addressing developmental, pedagogical, and ethical considerations, future research can contribute to a more nuanced, balanced, and sustainable model of AI-assisted foreign language education.

## 6. Conclusions

In conclusion, this study highlights the transformative potential of AI-assisted learning in foreign language education by foregrounding its role in shaping learner agency rather than merely improving instructional efficiency. The findings suggest positive associations between AI-assisted learning and multiple dimensions of learner agency, including autonomy, engagement, motivation, self-efficacy, enjoyment, and willingness to communicate, although these associations vary substantially across contexts. The extremely high level of heterogeneity across studies underscores that the effects of AI-assisted learning on learner agency are highly context-dependent rather than uniform; notably, meta-regression analysis indicates that AI tool type alone cannot account for this variability, emphasizing the priority of pedagogical integration over simple technological categorization. Consequently, current evidence does not justify strong claims regarding the consistent effectiveness of AI in enhancing foreign language learner agency, but instead highlights the contingent nature of these relationships, necessitating more theoretically precise and methodologically controlled research before generalizable conclusions can be drawn.

## Figures and Tables

**Table 1:** Coding Table.

No.	Study Name	Journal	Sample Size	Subgroup	First Language	Educational Level	AI Type	Effect Size
1	Shi (2024)	Journal of Educational Computing Research	274	Autonomy	Chinese	University	general AI-assisted	0.912
2	Guo and Wang (2025)	European Journal of Education	96	Engagement	Chinese	University	general AI-assisted	0.736
				Engagement				0.484
				Engagement				0.666
				Engagement				0.516
3	Z. Liu et al. (2024)	Computer Assisted Language Learning	65	Self-Efficacy	Chinese	Elementary school	general AI-assisted	0.525
				Enjoyment				0.434
4	Kim and Su (2024)	SYSTEM	65	Willingness to Communicate	Korean	Elementary school	AI chatbot	0.89
5	Qiao and Zhao (2023)	Frontier in Psychology	93	Autonomy	Chinese	University	AI-Integrated Learning Platforms	0.296
6	Shen et al. (2023)	Frontier in Psychology	42	Engagement	Chinese	University	general AI-assisted	0.33
				Engagement				0.414
7	Zheng et al. (2025)	Computer Assisted Language Learning	83	Self-Efficacy	Chinese	University	general AI-assisted	0.267
8	Song and Song (2023)	Frontier in Psychology	50	Motivation	Chinese	University	general AI-assisted	0.438
9	C. Zhang et al. (2024)	SYSTEM	113	Willingness to Communicate	Chinese	University	AI-Integrated Learning Platforms	0.12
10	Mohammed and Khalid (2025)	Language Testing in ASIA	322	Motivation	Kurdish	University	general AI-assisted	0.592
				Enjoyment				0.062
11	Chen (2025)	Interactive Learning Environments	50	Self-Efficacy	Chinese	University	AI chatbot	0.424
				Motivation				0.608
12	Wei (2023)	Frontier in Psychology	60	Motivation	Chinese	University	general AI-assisted	0.151
				Autonomy				0.487
13	G. L. Liu et al. (2024b)	European Journal of Education	299	Motivation	Chinese	University	AI-IDLE	0.5
				Enjoyment				0.26
14	J. Xu and Liu (2025)	Learning and Motivation	81	Motivation	Chinese	University	AI chatbot	0.443
				Enjoyment				0.478
				Autonomy				0.441
				Motivation			AI-Integrated Learning Platforms	0.438
				Enjoyment				0.414
				Autonomy				0.585
15	G. L. Liu et al. (2024a)	Computers in Human Behavior	690	Motivation	Chinese	University	AI-IDLE	0.88
				Enjoyment				0.77
16	Namaziandost and Rezai (2024)	SYSTEM	398	Enjoyment	Persian	University	AI-Integrated Learning Platforms	0.863
				Autonomy				0.421
				Motivation				0.624
				Autonomy				0.578
17	J. H. Lee et al. (2023)	RELC Journal	120	Enjoyment	Korean	Elementary school	general AI-assisted	0.194
18	Azamatova et al. (2023)	InternationalL Journal of Education in Mathmatics Science and Technology	64	Motivation	Kazakh	University	general AI-assisted	0.631
19	J. S. Lee et al. (2024)	Journal of Multilingual and Multicultural Development	308	Enjoyment	Chinese	Secondary school	AI-IDLE	0.53
				Enjoyment				0.36
				Willingness to Communicate				0.51
				Willingness to Communicate				0.38
				Enjoyment				0.48
				Enjoyment				0.36
				Willingness to Communicate				0.47
				Willingness to Communicate				0.39
20	Yuan and Liu (2025)	Computers in Human Behavior	383	Motivation	Chinese	University	general AI-assisted	0.158
				engagement				0.145
				Enjoyment				0.219
21	J. Xu and Li (2024)	European Journal of Education	408	Engagement	Chinese	University	general AI-assisted	0.477
								0.718
								0.75
								0.649
22	C. Wang et al. (2024)	SYSTEM	66	Willingness to Communicate	Chinese	University	general AI-assisted	0.059
				Enjoyment				0.146
				Willingness to Communicate				0.504
				Enjoyment				0.289
23	Ebadi and Amini (2024)	Interactive Learning Environments	256	Motivation	Persian	University	general AI-assisted	0.76
				Motivation				0.3
24	Ouyang et al. (2024)	International Review of Research in Open and Distributed Learning	80	Engagement	Chinese	University	general AI-assisted	0.538
				Willingness to Communicate				0.73
25	Tai and Chen (2020)	Interactive Learning Environments	112	Willingness to Communicate	Chinese	Secondary school	general AI-assisted	0.89
26	Rad et al. (2023)	Interactive Learning Environments	46	Engagement	Persian	University	general AI-assisted	0.998
27	Hapsari et al. (2023)	Language Teaching Research Quarterly	18	Self-Efficacy	Arabic	University	general AI-assisted	0.643
28	S. Wang et al. (2023)	Education and Information Technologies	561	Self-Efficacy	Chinese	University	general AI-assisted	0.515
29	J. H. Lee et al. (2023)	RELC JOURNAL	121	Enjoyment	Korean	Elementary school	AI-IDLE	0.0896
				Enjoyment				0.1435
30	Huang et al. (2025)	International Journal of Applied Linguistics	147	Engagement	Japanese	University	AI chatbot	0.2
								0.283
				Self-efficacy				0.604
31	Mei (2025)	Interactive Learning Environments	728	Self-Efficacy	Chinese	University	general AI-assisted	0.530
				Willingness to communicate				0.536
32	Zou et al. (2025)	RECALL	359	Enjoyment	Pakistani	University	AI-IDLE	0.34

**Table 2:** Subgroup Analysis.

Groups	Number Studies (K)	Effect Size and 95% Interval			Heterogeneity				Tau-Squared	
		Point Estimate (r)	Lower Limit	Upper Limit	Q	df (Q)	*p*	I^2^	Tau^2^	Tau
Autonomy	7	0.581	0.305	0.767	233.491	6	<0.001	97.430	0.214	0.462
Engagement	15	0.648	0.476	0.773	634.252	14	<0.001	97.793	0.243	0.493
Enjoyment	18	0.392	0.226	0.535	621.849	17	<0.001	97.266	0.151	0.389
Motivation	13	0.540	0.344	0.691	515.555	12	<0.001	97.672	0.196	0.442
Self-Efficacy	7	0.510	0.447	0.568	10.945	6	0.090	45.179	0.005	0.067
Willingness to Communicate	11	0.557	0.418	0.671	190.336	10	<0.001	94.746	0.088	0.297
Overall	71	0.517	0.469	0.562	2325.001	70	<0.001	96.989	0.148	0.385

**Table 3:** First Language Analysis.

Groups	Number Studies (K)	Effect Size and 95% Interval			Heterogeneity				Tau-Squared	
		Point Estimate (r)	Lower Limit	Upper Limit	Q	df (Q)	*p*	I^2^	Tau^2^	Tau
Chinese	52	0.505	0.429	0.574	1373.427	51	<0.001	96.287	0.119	0.345
Persian	7	0.814	0.612	0.916	583.257	6	<0.001	98.971	0.325	0.570
Korean	4	0.427	−0.066	0.752	86.608	3	<0.001	96.536	0.274	0.523
Japanese	3	0.378	0.098	0.603	20.224	2	<0.001	90.111	0.063	0.252
Kurdish	2	0.355	−0.230	0.752	60.846	1	<0.001	98.357	0.188	0.433
Kazakh	1	0.631	0.455	0.759	-	-	-	-	-	-
Pakistani	1	0.340	0.245	0.428	-	-	-	-	-	-
Arabic	1	0.643	0.252	0.854	-	-	-	-	-	-

**Table 4:** Educational Level Analysis.

Groups	Number Studies (K)	Effect Size and 95% Interval			Heterogeneity				Tau-Squared	
		Point Estimate (r)	Lower Limit	Upper Limit	Q	df (Q)	*p*	I^2^	Tau^2^	Tau
Elementary school	6	0.444	0.113	0.686	90.500	5	<0.001	94.475	0.194	0.441
Secondary school	9	0.511	0.396	0.610	110.654	8	<0.001	92.770	0.046	0.213
University	56	0.546	0.465	0.618	2045.891	55	<0.001	97.312	0.165	0.406

**Table 5:** Meta-regression results for education level as a moderator of AI-assisted learning effects on learner agency.

Moderator	Coefficient (β)	SE	95% CI		Z	*p*
			(Lower)	(Upper)		
Primary school	0.4752	0.1631	0.1556	0.7947	2.91	0.0018
Secondary school	0.096	0.2083	−0.3123	0.5042	0.46	0.3225
University	0.1372	0.1714	−0.1988	0.4733	0.8	0.2117
Model statistics:						
Q_model_ = 0.68, df = 2, *p* = 0.7111						
τ^2^ = 0.1473, I^2^ = 96.97%						
R^2^ analog = 0.01						
Number of studies = 71						

Note. Effect sizes were transformed to Fisher’s Z values. Elementary school was used as the reference category. Q_model_ represents the omnibus test of moderators. τ^2^ and I^2^ indicate residual heterogeneity.

**Table 6:** Meta-regression results for AI Type as a moderator of AI-assisted learning effects on learner agency.

Moderator	Coefficient (β)	SE	95% CI		Z	*p*
			(Lower)	(Upper)		
general AI-assisted	0.5783	0.1368	0.3101	0.8465	4.23	<0.001
AI chatbot	−0.0721	0.1713	−0.4079	0.2636	−0.42	0.3368
AI-IDLE	−0.0014	0.192	−0.3776	0.3748	−0.01	0.4972
AI-Integrated Learning Platforms	0.0631	0.152	−0.2349	0.361	0.41	0.3391
Model statistics:						
Q_model_ = 1.27, df = 3, *p* = 0.7372						
τ^2^ = 0.1556, I^2^ =97.09%						
R^2^ analog = 0.00						
Number of studies = 71						

Note. Effect sizes were transformed to Fisher’s Z values. Elementary school was used as the reference category. Q_model_ represents the omnibus test of moderators. τ^2^ and I^2^ indicate residual heterogeneity.

## Data Availability

Data is contained within the article.
